# EEG microstate analysis between patients with major depressive disorder, subclinical depression, and healthy controls

**DOI:** 10.3389/fpsyt.2025.1707099

**Published:** 2025-11-26

**Authors:** Jinwon Chang

**Affiliations:** 1 Williams College, Williamstown, MA, United States; 2 Department of Psychiatry, Beth Israel Deaconess Medical Center, Harvard Medical School, Boston, MA, United States

**Keywords:** Electroencephalography, microstate, depression, major depressive disorder, reliability

## Abstract

**Introduction:**

Electroencephalography (EEG) microstates have emerged as potential biomarkers of large-scale brain network dynamics. However, their role in depression remains unclear due to inconsistent findings and limited replication. This study investigated whether microstate parameters can differentiate depression.

**Methods:**

Resting-state EEG was analyzed from 122 young adults [76 controls, 23 subclinical depression, 23 major depressive disorder (MDD)]. Microstate analysis was conducted using standardized pipelines (MICROSTATELAB in EEGLAB), with duration, occurrence, and coverage extracted for six-class solutions. Group differences were assessed using Bayesian ANCOVA, while associations with depressive and anxiety symptoms (BDI, STAI) were evaluated with Bayesian regression. Replication analyses were performed using a second independent EEG recording. Test–retest reliability was assessed with intraclass correlation coefficients.

**Results and discussion:**

Microstate G duration was reduced in both subclinical and high-symptom groups compared with controls and showed negative associations with BDI and STAI scores. These effects are partially replicated in the second dataset. Microstate G also demonstrated high test–retest reliability (ICC=0.842). In contrast, microstate A showed weaker and less reliable associations with depressive symptoms. Microstate G represents a reliable electrophysiological marker of depressive symptomatology. These findings highlight EEG microstate analysis as a promising approach for developing objective, dimensional biomarkers of depression.

## 1 Introduction

Depression is one of the most prevalent mental disorders, profoundly affecting mood, cognition, and behavior. According to the World Health Organization (1), more than 300 million individuals worldwide are currently living with depression. The recent COVID-19 pandemic has further exacerbated global rates of depression, anxiety, and stress (2). Importantly, depressive episodes often recur across the lifespan, imposing long-term cognitive and financial burdens (3). Despite its high prevalence and chronicity, the clinical diagnosis of depression still relies heavily on subjective assessments and physician judgment. Efforts to operationalize depression as a dimensional construct have proven even more challenging, underscoring the need for reliable biological markers that could facilitate early detection and intervention.

Neuroimaging modalities such as magnetic resonance imaging (MRI) and electroencephalography (EEG) have been explored as alternatives to purely clinical assessments. Structural MRI, functional MRI, and diffusion tensor imaging have shown potential in identifying neurobiological markers of major depressive disorder (MDD) (4). However, these methods are costly and limited in their applicability for early-stage diagnosis. By contrast, EEG offers a low-cost, noninvasive, and widely accessible means to assess brain function. Yet, traditional EEG markers in depression remain inconsistent. Reported abnormalities in spectral power are often conflicting (5), functional connectivity has demonstrated poor test–retest reliability (6), and spectral analyses, particularly in the gamma band, are complicated by methodological issues in baseline correction and broadband activity (7).

Against this background, EEG microstate analysis has emerged as a promising approach for characterizing large-scale brain dynamics in resting state (8). Microstates are quasi-stable scalp topographies that persist for tens of milliseconds before rapidly transitioning to a different state, thought to reflect the sequential activation of large-scale neural networks (9). Each canonical microstate type (e.g., A–D) is associated with distinct temporal features, spatial topographies, and putative functional correlates (10). Crucially, microstate parameters such as duration, occurrence, and coverage exhibit high test–retest reliability across short- and long-term intervals, different electrode montages, and clustering procedures, reinforcing their utility as potential biomarkers (11).

EEG microstates have already been applied to characterize several psychiatric and neurological conditions (10). Robust alterations have been demonstrated in schizophrenia (12, 13), and recent meta-analyses report consistent though smaller effects in mood and anxiety disorders (14). Specifically, increased occurrence of microstate B (Hedges’ g = 0.41) and decreased occurrence of microstate D (g = –0.34) have been associated with mood disorders. Nonetheless, findings in depression remain heterogeneous. For example, Cao et al. (15) reported a reduction in microstate B occurrence in both subclinically depressed patients and those with comorbid insomnia relative to controls, while Murphy et al. (16) observed reductions in microstate D coverage, duration, and occurrence in MDD, correlating with symptom severity. In contrast, Damborská et al. (17) found no group-level differences but did report significant associations between microstate A and depressive symptoms.

These inconsistencies highlight the limited and heterogeneous state of the current literature, emphasizing the need for standardized methods and replication across independent datasets. To address this gap, the present study applies a standardized microstate analysis pipeline (MICROSTATELAB within EEGLAB; 18) to extract microstate maps and quantitative features using only those temporal parameters demonstrated to be highly reliable (11). Critically, two independent EEG recordings from the same subjects were analyzed to evaluate short-term replicability of each microstate parameter. Finally, two different categorical (MDD vs. subclinical depression vs. healthy controls and high depressive symptomatology vs. healthy controls) frameworks were applied, enabling a more comprehensive characterization of depression-related alterations in EEG microstates. Therefore, the current study aims to investigate the differences in EEG microstate parameters between depressed and healthy individuals using standardized and replicable methods.

## 2 Methods

### 2.1 Participants

Data were obtained from a publicly available OpenNeuro dataset (19), originally collected to investigate EEG correlates of depression and anxiety during a behavioral task (20). Participants were university students aged 18–25 years, with no history of seizures or head trauma and no current use of psychoactive medications. A total of 76 healthy controls (CTL) and 46 individuals with high depressive symptomatology (DEP) were included. The DEP group comprised 23 subclinical participants with depressive symptoms and 23 participants with past or current MDD. Healthy controls were required to demonstrate consistently low Beck Depression Inventory (BDI) scores (<7) across two assessments (2–14 weeks apart), report no history of MDD, and show no indication of an Axis I disorder as screened by the computerized Electronic Mini International Neuropsychological Interview (eMINI). Individuals in the DEP group were required to maintain consistently elevated BDI scores (≥13) across the same interval. DEP participants were invited to complete a Structured Clinical Interview for Depression ($20/hour compensation). Among them, 14 declined the interview, 9 did not meet criteria for MDD, 12 met criteria for past MDD, and 11 met criteria for current MDD. DEP participants (BDI ≥13) without past or current MDD were classified as subclinical depression group, which is consistent with previous studies that treated groups with high depression scores (e.g., BDI) but without clinical interview as subclinical depression (15). Previous reports on these data (21) classified only current and past MDD participants (N = 21) as depressed. The present study extends this approach by applying two categorical (CTL vs. subclinical vs. MDD and DEP vs CTL) analyses. All participants provided written informed consent in accordance with the University of Arizona’s ethical guidelines.

### 2.2 Experimental procedure

Each participant completed two EEG sessions: one before and one after a probabilistic learning task (20). In each session, participants first completed the BDI and the Spielberger Trait Anxiety Inventory (STAI), followed by six minutes of resting-state EEG. The resting-state protocol alternated with 1-minute eyes-open (O) and eyes-closed (C) blocks (The ordering of the blocks was either OCCOCO or COOCOC, randomly assigned for each participant). The second EEG session, following the task, included one or two eyes-closed blocks (OCO or COC). Only eyes-closed data were analyzed for microstate computation because eyes-opened data could introduce significant eye artifacts. Two participants were excluded due to unstable BDI assessments or missing EEG data across both sessions.

### 2.3 EEG acquisition and preprocessing

EEG was recorded with a 64-channel Synamps2 system (Compumedics Neuroscan) using the 10/10 electrode placement system. Signals were sampled at 500 Hz with a 0.5–100 Hz bandpass filter, and electrode impedance was maintained below 10 kΩ. Data were referenced to a midline electrode located between Cz and CPz.

Preprocessing was performed in EEGLAB (22). Non-EEG channels (e.g., EOG, EKG) were removed, and only eyes-closed segments were retained by excluding pre-labeled non-EEG electrodes and eyes-opened segments. Signals were filtered at 2–20 Hz in accordance with MICROSTATELAB recommendations (18) and microstate reliability findings (11). Artifact Subspace Reconstruction (ASR) was applied to remove artifacts. Flat channels (≥5 s) or channels with high-frequency noise >4 SD above the mean were removed. Burst artifacts were detected with a 0.5 s sliding window and a 20 SD threshold. Bad data segments were excluded based on RMS thresholds (−lnf7) with a 25% channel outlier limit. Following average referencing, independent component analysis (ICA) was performed, and components reflecting ocular or muscle artifacts (≥70% probability) were rejected.

### 2.4 EEG microstate analysis

Microstate analysis followed the MICROSTATELAB pipeline (18). Individual microstate templates were identified using a k-means clustering algorithm (20 restarts) applied to global field power (GFP) peaks, with polarity ignored. Clustering was performed for 4–7 classes. Group-level mean maps were then computed, followed by a grand mean microstate map across all groups. Sorting of maps was conducted using the EEG microstate metamap (23). The optimal number of classes was determined as the solution achieving >75% shared variance between the grand mean maps and established template maps. Only the six-class solution met this criterion and was retained for further analysis. Grand mean maps from the six-class solution were then used to sort individual maps. GFP peaks and grand mean templates were applied for backfitting to compute temporal parameters (Duration: mean length of each microstate type; Occurrence: average number of occurrences per second; Coverage: percentage of total EEG occupied by each microstate). Transition probabilities were not analyzed, given their poor test–retest reliability (11).

### 2.5 Statistical analysis

All statistical analyses were conducted in MATLAB R2024b (MathWorks, Natick, MA) and MedCalc v20.218 (MedCalc Software Ltd., Ostend, Belgium). Two comparison frameworks were employed: CTL versus DEP, and CTL versus subclinical versus MDD. Clinical and demographic variables, including BDI, STAI, and age, were compared using Kruskal–Wallis tests with Dunn’s *post hoc* correction, while sex distributions were assessed with chi-square tests. Such statistical analyses on clinicodemographic variables were conducted to confirm the clinical difference between depressed and non-depressed individuals and importance of controlling covariables. Group-level differences in microstate parameters were examined using Bayesian ANCOVA with sex and age included as a covariate. Linear regression models, also controlling for sex and age, were used to examine the relationship between microstate parameters and BDI or STAI scores. Bayes factors were interpreted according to Kass and Raftery (24), with BF10 values between 3 and 10 considered moderate evidence and values greater than 10 considered strong evidence. To assess replicability, all analyses were repeated using the second EEG dataset, which consisted of shorter eyes-closed segments recorded after the behavioral task. Test–retest reliability of microstate temporal parameters was evaluated across the two sessions using intraclass correlation coefficients (ICC (2,k), two-way random effects, absolute agreement, average measures), following the guidelines of Koo and Li (25).

## 3 Results

Clinical and demographic characteristics of each group are presented in **Table 1**. There were no significant differences in age across groups, but sex distributions differed significantly between CTL and both DEP and subclinical/MDD groups (p < 0.0001). As expected, both BDI and STAI scores were substantially higher in the subclinical, MDD, and DEP groups compared with controls (p < 0.001).

**Table 1:** Clinical demographic characteristics of each group.

	CTL	Subclinical	MDD	DEP	p value
N	74	23	23	46	
age	19.0 (1.2)	18.6 (0.9)	18.9 (1.3)	18.7 (1.1)	0.237^a^/0.225^b^
sex	39	19	15	34	<0.0001^a, b^
BDI	1.8 (1.7)	22.8 (4.1)	21.7 (5.6)	22.2 (4.9)	<0.001^a, b^
STAI	31.1 (5.5)	55.7 (7.3)	55.9 (7.0)	55.8 (7.1)	<0.001^a, b^

a CTL vs subclinical and CTL vs MDD.

b CTL vs DEP.

**Figure 1:**
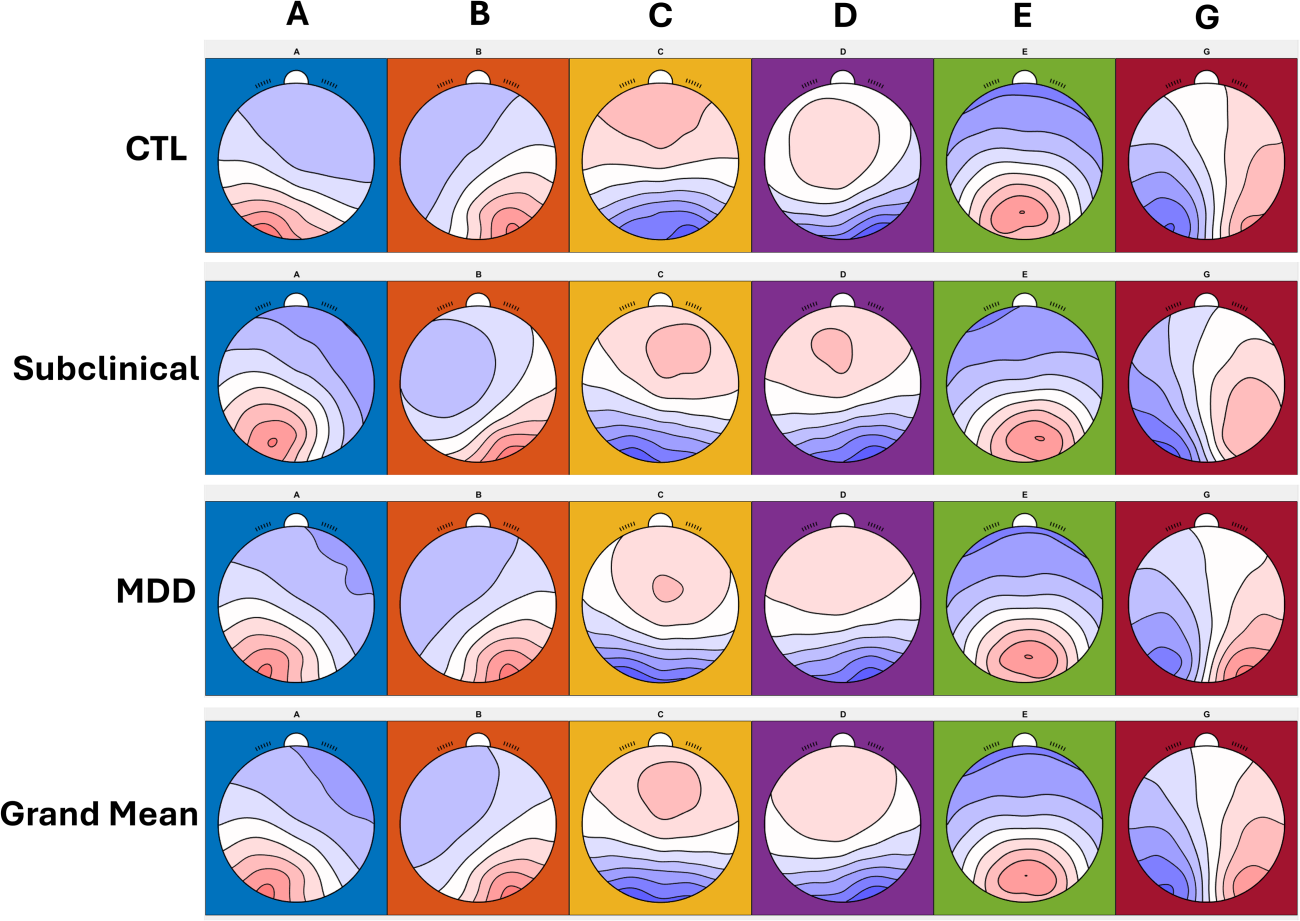
EEG microstate maps of each group with 6 clustering classes. Six microstates were labeled through **A-G** within and across the groups.

Six microstate classes (A, B, C, D, E, and G) were identified across participants, consistent with published templates (23). Group-averaged topographies are shown in **Figure 1**.

**Figure 2:**
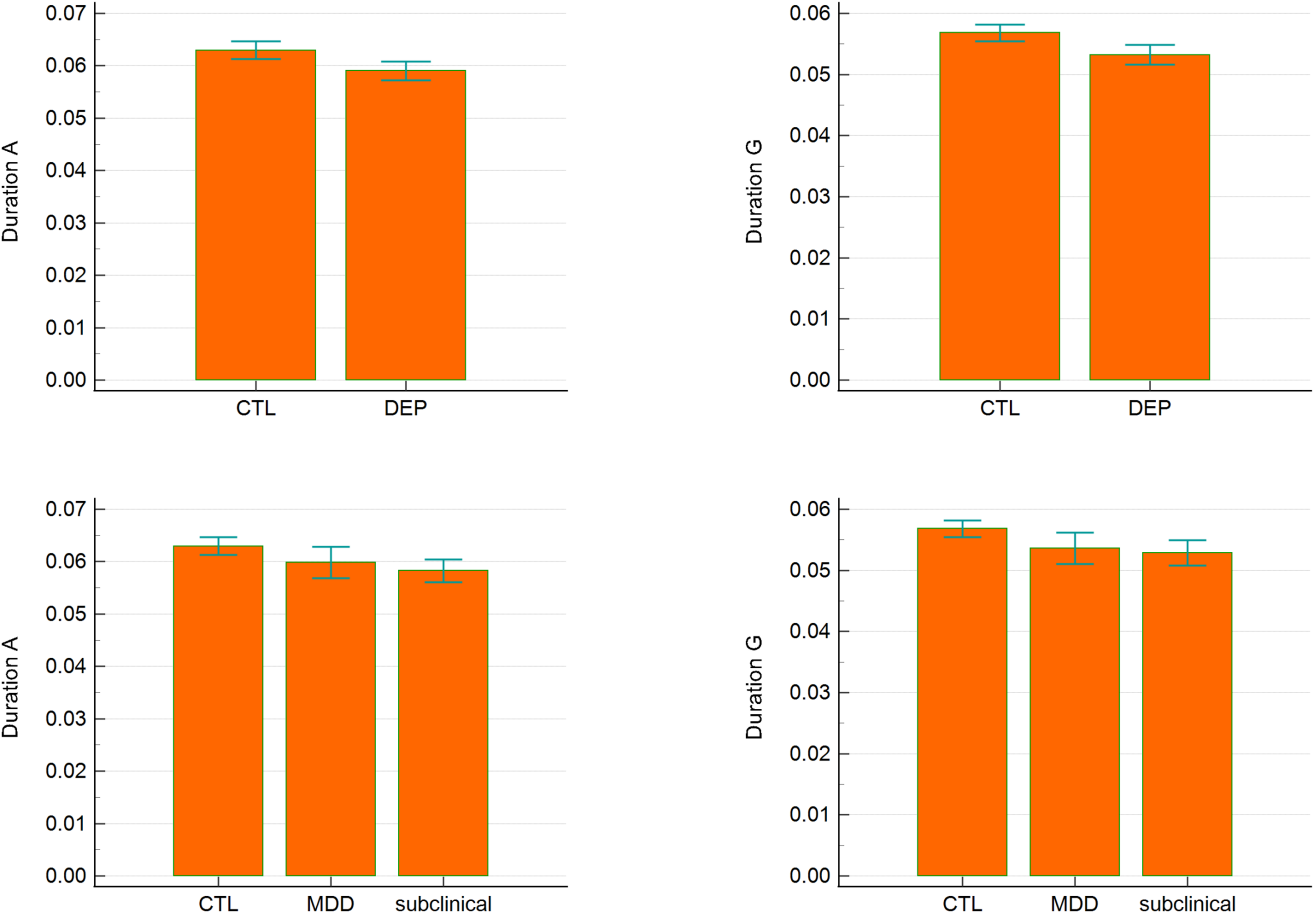
Duration of microstates A and G across groups. Error bars represent 95% confidence intervals.

Bayesian ANCOVA revealed moderate evidence for group effects in the duration of microstates A (BF10M = 3.135) and G (BF10M = 3.679). *Post hoc* comparisons indicated strong evidence for differences between CTL and the subclinical group for both microstates (posterior odds = 6.4, BF10 = 10.896 for duration A; posterior odds = 5.905, BF10 = 10.053 for duration G). No significant differences were observed between CTL and MDD or between subclinical and MDD. In two-group comparisons (CTL vs. DEP), there was strong evidence for longer durations of microstates A and G in CTL relative to DEP (BF10 = 16.925 and BF10 = 31.121, respectively). Other temporal parameters, including occurrence and coverage, did not differ significantly between groups (**Table 2**, **Figure 2**).

**Table 2:** Group differences in temporal parameters of microstates.

Duration	BF10_M_	CTL VS subclinical	CTL VS MDD	Subclinical VS MDD	BF10_M_	CTL VS DEP
A	3.135	6.4(10.896)	0.682(1.162)	0.237(0.404)	7.448	16.925
B	0.78				1.807	
C	0.455				0.79	
D	0.44				1.023	
E	0.94				2.238	
G	3.679	5.905(10.053)	1.408(2.396)	0.187(0.319)	9.663	31.121
Occurrence						
A	0.181				0.401	
B	0.624				1.499	
C	0.373				0.824	
D	0.452				1.094	
E	0.104				0.215	
G	0.2				0.433	
Coverage						
A	0.102				0.203	
B	0.118				0.242	
C	0.109				0.203	
D	0.106				0.213	
E	0.142				0.299	
G	0.105				0.195	

For *post hoc* comparisons, posterior odds (BF10, uncorrected) were reported after the multiple correction by fixing 0.5 to the prior probability that the null hypothesis holds across all comparisons. Individual comparisons were based on the default t-test using Cauchy [0, r = 1/sqrt(2)] prior.

Bayesian linear regression revealed moderate evidence that both microstate A (BF10 = 3.312) and G (BF10 = 5.227) durations were negatively associated with BDI scores (**Table 3**). The posterior mean coefficients were –274.92 (95% CI: –526.49 to –23.36) for microstate A and –359.86 (95% CI: –659.60 to –60.12) for microstate G, indicating that shorter durations were associated with higher depressive symptom severity. For anxiety symptoms (STAI), there was anecdotal evidence for an association with microstate A (BF10 = 2.236), but strong evidence for an association with microstate G (BF10 = 12.715). The posterior mean coefficient for microstate G was –531.07 (95% CI: –911.84 to –150.30), suggesting that reduced microstate G duration was linked to increased trait anxiety.

**Table 3:** Bayesian regression analyses of microstates A and G predicting BDI and STAI scores.

	Variables	BF10_M_	R²	Posterior coefficient
BDI	Duration A	3.312	0.077	-274.92 (-526.49, -23.36)
	Duration G	5.227	0.085	-359.86 (-659.60, -60.12)
STAI	Duration A	2.236	0.081	-321.45 (-643.27, 0.37)
	Duration G	12.715	0.112	-531.07 (-911.84, -150.30)

Posterior coefficients were reported as a mean estimate (95% confidence interval).

Further analyses were applied on Duration A and G on the second dataset that was recorded after the cognitive task.

Analyses were repeated using the shorter eyes-closed recordings obtained after the cognitive task. In this dataset, evidence for group differences was weaker (**Table 4**). For three-group comparisons, only anecdotal evidence supported differences in microstate A and G durations. In two-group comparisons (CTL vs. DEP), anecdotal evidence was observed for microstate A (BF10M = 0.641), while moderate evidence remained for microstate G (BF10M = 5.67; BF10 = 11.299 for CTL vs. DEP).

**Table 4:** Group differences in duration A and G in the second dataset.

Variable	BF10_M_	CTL VS subclinical	CTL VS MDD	subclinical VS MDD	BF10_M_	CTL VS DEP
Duration A	0.368	0.585 (0.996)	0.173 (0.294)	0.231 (0.393)	0.641	0.569
Duration G	2.176	2.929 (4.987)	0.935 (1.591)	0.181 (0.308)	5.67	11.299

For *post hoc* comparisons, posterior odds (BF10, uncorrected) were reported after the multiple correction by fixing 0.5 to the prior probability that the null hypothesis holds across all comparisons. Individual comparisons were based on the default t-test using Cauchy [0, r = 1/sqrt(2)] prior.

Dimensional analyses in the second dataset showed anecdotal evidence for associations between microstate A duration and both BDI and STAI scores (**Table 5**). For microstate G, moderate evidence supported its association with BDI (BF10 = 5.571, posterior mean coefficient = –256.88, 95% CI: –508.29 to 0), while only anecdotal evidence was observed for STAI (BF10 = 2.302).

**Table 5:** Linear regression on BDI and STAI score using Duration A and G in the second dataset.

	Variables	BF10_M_	R²	Posterior coefficient
BDI	Duration A	0.631	0.046	-35.79 (-201.32, 55.26)
	Duration G	5.571	0.086	-256.88 (-508.29, 0)
STAI	Duration A	0.348	0.047	-10.23 (-192.15, 80.48)
	Duration G	2.302	0.082	-224.45 (-575.20, 1.612)

Posterior coefficients were reported as a mean estimate (95% confidence interval).

Test–retest reliability was assessed across the two EEG sessions (**Table 6**). Microstate G duration demonstrated good reliability (ICC = 0.842, 95% CI: 0.773–0.890), whereas microstate A duration showed poor reliability (ICC = 0.616, 95% CI: 0.326–0.766).

**Table 6:** Intraclass correlation coefficients of the duration of microstate A and G over two repeated sessions.

	Average measures	95% confidence interval
Duration G	0.842	0.773 to 0.890
Duration A	0.616	0.326 to 0.766

## 4 Discussion

The present study demonstrates that EEG microstate dynamics, particularly the duration of microstate G, are associated with depressive symptomatology. Although microstate A also showed group-level differences and moderate associations with depressive severity, its low test–retest reliability and inconsistent replication across datasets limit its potential as a robust biomarker. In contrast, microstate G displayed consistent group differences, replicated associations with depressive symptoms, and strong test–retest reliability, supporting its candidacy as a reliable electrophysiological marker of depression.

Microstate A distinguished controls from the subclinical group in the primary dataset and showed moderate associations with BDI scores. However, these effects did not replicate in the second dataset, which was limited by shorter recording duration, and reliability analysis indicated only poor stability across sessions. These findings suggest that while microstate A may reflect depression-related alterations, its utility is constrained by methodological sensitivity and inconsistency.

Microstate G demonstrated more robust and reproducible results. It differentiated both DEP and subclinical groups from controls, was negatively associated with both depressive and anxiety symptom severity and showed strong test–retest reliability. Although replication in the second dataset yielded only moderate evidence for group differences and associations with BDI scores, the short recording length likely reduced parameter stability. Taken together, these findings indicate that microstate G is a promising biomarker for depression-related network alterations.

Importantly, the present results emphasize the value of a dimensional perspective. Microstate G consistently distinguished individuals with elevated depressive symptoms from healthy controls, yet no significant differences emerged between subclinical and MDD groups. Furthermore, microstate G duration was linearly associated with depressive severity across participants, supporting the view that depression may be better characterized as a continuous spectrum rather than as discrete diagnostic categories. This interpretation aligns with prior findings demonstrating that microstate alterations are more strongly linked to symptom dimensions than to categorical diagnoses (17).

The dimensional specificity of our findings is consistent with converging evidence from other neuroimaging modalities. For example, Yang et al. (26) showed that functional connectivity between the posterior parietal thalamus and medial frontal gyrus was enhanced in subclinical depression compared with controls but reduced in MDD, indicating that network alterations are not linearly progressive across diagnostic categories. Similarly, Eggart et al. (27) reported impaired interoceptive accuracy predominantly in moderate depression, while other studies have documented distinct neural alterations in subclinical versus MDD populations (28–30). Our finding that microstate G differs in subclinical but not MDD groups reinforces the notion that subclinical depression represents a distinct neurophysiological state, rather than a mere precursor to MDD. At the same time, the ability of microstate G to separate high-symptom groups (DEP) from controls suggests that categorical distinctions retain clinical value when dimensional severity is considered.

The neurophysiological interpretation of microstate G remains less established than for canonical microstates A–D. In the present study, microstate G consistently displayed a bilateral temporal–occipital topography, which may correspond to posterior default mode network (DMN) activity (31). Although direct evidence linking microstate G to large-scale functional networks is limited, its reproducibility across studies (23) and its robust association with depressive symptomatology in the present work underscore the importance of further investigating its neural correlates. Especially, as altered posterior DMN activity is related to major depressive disorders (4), its relationship with microstate G might be important to establish functional associations between depression and microstate G. This approach with various number of microstate maps over only four A-D maps would be significant for identifying accurate functional roles of EEG microstate maps over numerous interconnected functional networks.

Several limitations should be acknowledged. First, the dataset consisted of young adults aged under 25 years, whereas depression is most prevalent between the ages of 25 and 65 (32). Therefore, further investigation with prospective study design is needed for how these young, depressed individuals might develop their symptoms into bipolar disorder, MDD, or other comorbidities related to EEG microstates Second, the modest sample size and restricted age range may have reduced generalizability. Third, although we employed standardized pipelines and replicated analyses across two datasets, the second dataset consisted of shorter recordings, which may have reduced microstate stability. Also, the current study’s participants did not take any medication, so further study is needed to investigate whether drug use or drug resistance might affect the microstate configuration or temporal dynamics. Future research should extend these findings to larger, more diverse populations, incorporate longer recording periods, and combine microstate analysis with complementary neuroimaging techniques to establish stronger neurophysiological interpretations.

In sum, the present study provides evidence that EEG microstate duration, particularly microstate G, is associated with depression both dimensionally and categorically. While microstate A showed preliminary associations, microstate G emerged as the more reliable and replicable marker, differentiating individuals with elevated depressive symptoms from controls and correlating with symptom severity. These findings highlight the potential of EEG microstate analysis to contribute to the identification of objective biomarkers of depression and emphasize the importance of integrating dimensional approaches in psychiatric research.

## 5 Conclusions

This study investigated the utility of EEG microstate parameters in characterizing depression through both two categorical frameworks. The results demonstrated that the duration of microstate G consistently differentiated individuals with elevated depressive symptoms from healthy controls and showed strong associations with both depressive and anxiety severity. Importantly, microstate G also exhibited high test–retest reliability across sessions, underscoring its robustness as a potential biomarker.

Although microstate A showed preliminary associations with depressive symptoms, its limited reliability and inconsistent replication suggest that it is less suitable as a diagnostic marker. In contrast, microstate G emerged as a more stable and reproducible indicator of depression-related alterations in large-scale brain network dynamics. These findings support the integration of EEG microstate analysis into biomarker development for depression and highlight the importance of adopting dimensional approaches that capture symptom severity beyond categorical diagnoses.

## Data Availability

The original contributions presented in the study are included in the article/supplementary material. Further inquiries can be directed to the corresponding author.
